# Lesser-Consumed Tropical Fruits and Their by-Products: Phytochemical Content and Their Antioxidant and Anti-Inflammatory Potential

**DOI:** 10.3390/nu14173663

**Published:** 2022-09-05

**Authors:** Beatriz Haydee Belmonte-Herrera, J. Abraham Domínguez-Avila, Abraham Wall-Medrano, J. Fernando Ayala-Zavala, Alejandra M. Preciado-Saldaña, Norma J. Salazar-López, Leticia X. López-Martínez, Elhadi M. Yahia, R. Maribel Robles-Sánchez, Gustavo A. González-Aguilar

**Affiliations:** 1 Centro de Investigación en Alimentación y Desarrollo A. C., Carretera Gustavo Enrique Astiazarán Rosas No. 46, Col. La Victoria, Hermosillo 83304, Mexico; 2 CONACYT-Centro de Investigación en Alimentación y Desarrollo A. C., Carretera Gustavo Enrique Astiazarán Rosas No. 46, Col. La Victoria, Hermosillo 83304, Mexico; 3 Instituto de Ciencias Biomédicas, Universidad Autónoma de Ciudad Juárez, Ciudad Juárez 32310, Mexico; 4 Facultad de Medicina de Mexicali, Universidad Autónoma de Baja California, Dr. Humberto Torres Sanginés S/N, Centro Cívico, Mexicali 21000, Mexico; 5 Facultad de Ciencias Naturales, Universidad Autónoma de Querétaro, Avenida de las Ciencias S/N, Juriquilla, Querétaro 76230, Mexico; 6 Departamento de Investigación y Posgrado en Alimentos, Universidad de Sonora, Hermosillo 83000, Mexico

**Keywords:** mamey, lychee, jackfruit, açaí, passion fruit, immunomodulatory, phenolics, antioxidants, bioactive compounds

## Abstract

Açaí, lychee, mamey, passion fruit and jackfruit are some lesser-consumed tropical fruits due to their low commercial production. In 2018, approximately 6.8 million tons of these fruits were harvested, representing about 6.35% of the total world production of tropical fruits. The present work reviews the nutritional content, profile of bioactive compounds, antioxidant and anti-inflammatory capacity of these fruits and their by-products, and their ability to modulate oxidative stress due to the content of phenolic compounds, carotenoids and dietary fiber. Açaí pulp is an excellent source of anthocyanins (587 mg cyanidin-3-glucoside equivalents/100 g dry weight, dw), mamey pulp is rich in carotenoids (36.12 mg β-carotene/100 g fresh weight, fw), passion fruit peel is rich in dietary fiber (61.16 g/100 dw). At the same time, jackfruit contains unique compounds such as moracin C, artocarpesin, norartocarpetin and oxyresveratrol. These molecules play an important role in the regulation of inflammation via activation of mitogen-activated protein kinases (including p38, ERK and JNK) and nuclear factor κB pathways. The properties of the bioactive compounds found in these fruits make them a good source for use as food ingredients for nutritional purposes or alternative therapies. Research is needed to confirm their health benefits that can increase their marketability, which can benefit the primary producers, processing industries (particularly smaller ones) and the final consumer, while an integral use of their by-products will allow their incorporation into the circular bioeconomy.

## 1. Introduction

The search for a healthier lifestyle has led to increased consumption of fruits, vegetables, and functional foods. Several studies indicate that diets rich in fruits and vegetables positively correlate with improved health, due to a reduced risk of cancer, obesity, inflammatory conditions, and cardiovascular diseases, in addition to their high nutritional value [1,2,3,4]. Fruits and vegetables are rich sources of fiber, vitamins, minerals, and bioactive compounds such as phenolic compounds, carotenoids, and betalains. Recently, these compounds and the foods that contain them have been the focal point of researchers due to their beneficial health effects [5,6].

Tropical fruits are regularly cultivated in the geographical zones that stretch from latitude 23°27′ N to 23°27′ S, while some can also be found at 37° N, such as in southern Spain. Temperatures in these areas differ, averaging 25 °C throughout the year and oscillating from 16 to 36 °C [7,8]. The economic value and popularity of tropical fruits are varied, as some are more known and consumed, while others are less known and less consumed [9]. Global production of main tropical fruits was approximately 100.2 million tons in 2018, with mango, pineapple, papaya, and avocado being the most important (52, 28, 14 and 6%, respectively), in addition to bananas (114 million tons in 2017). However, minor, less known, and less consumed tropical fruits are several, and some of the important ones, depending on the geographical zones, include jackfruit (*Artocarpus heterophyllus*), lychee (*Lychee chinensis*), passion fruit (*Passiflora edulis Sims F*. flavicarpa), açaí (*Euterpe oleraceae*) and mamey (*Pouteria sapota* (Jacq.) H. E. Moore & Stearn), which accounted for an annual production in 2017 of 3.7 million, 3.3 million, 1.5 million, 1.4 million and 14.66 million tons, respectively [10,11,12,13,14,15,16].

In contrast to the highly consumed fruits, lesser-consumed tropical fruits are not widely traded, but cultivated and consumed mostly locally or regionally. However, their economic and traditional values and consumption are very important in their original cultivation regions [9]. Only a minor percentage of the production of these lesser-consumed tropical fruits is traded in distant export markets; knowledge of these is scarce, and consumption is very low outside the areas where they are cultivated. Lately, their production and trade are gaining global importance, especially due to their health benefits. In producing regions, lesser-consumed tropical fruits play an important role, on food and nutrition security and as a source of income for local producers. Available household surveys from key producing areas indicate that the revenue from these fruits can account for up to 75 percent of the entire income of small rural households [14]. Thus, there is a need to focus on these fruits, which the present work aims to provide, while also considering the bioactive content of their byproducts, since they are minimally considered, as compared to the edible pulp.

Açaí grows in palms in the Brazilian Amazon and South America and is characterized by significant contents of anthocyanins, oleic acid, fiber, and phytosterols. In Brazil, the consumption of its juice is approximately 2 L per day [17]. Lychee is found mainly in Southeast Asia (particularly in China, Indonesia, Vietnam, and Thailand), where it is known by several names, including Chinese cherry, mountain lychee, or water lychee [18]. Mamey, a member of the *Sapotaceae* family, is native to Mexico, but tropical and subtropical cultivars can also be found in Central America and some Asian countries [19]. Passion fruit is native to Brazil, whose production is mainly used to produce juices and beverages, while its peel is used to produce flour and as a functional food ingredient [20]. Jackfruit, which belongs to the *Moraceae* family, is native to India but has been introduced to several tropical regions worldwide, including Mexico. Its fruits are large, reaching up to more than 50 kg, available all year, but production peaks are in June and December [21,22].

The health benefits of consuming the less-known tropical fruits are not fully understood, partly because they have been poorly studied. However, they have been correlated with interactions between their phytochemicals and key enzymes, cytokines and transcription factors involved in several signaling cascades. In addition, their effects on the antioxidant system are also significant since they can maintain and re-establish homeostasis [5,11,23,24].

It has been reported that by-products from lesser-consumed tropical fruits have several bioactive compounds, such as açaí seed, which can prevent weight gain, adiposity, and dyslipidemia, according to in vivo data (male mice, C57BL/6) [25]. Lychee peel has considerably higher amounts of phenolic compounds and scavenging capacity than other parts of the fruit. At the same time, its seeds prevent the growth of cancerous cells, in addition to antihyperglycemic, antihyperlipidemic, antiplatelet, and antiviral activities [26,27]. Jackfruit peel extracts have shown a high total flavonoid content, which correlates with its high antioxidant activity (AOXA) against the 2,2-diphenyl-1-picrylhydrazyl (DPPH) radical [28].

Using different by-products from lesser-known tropical fruits as food ingredients is an area of interest for further research. In this sense, passion fruit peel (PFP) supplementation is considered an important source of dietary fiber. Studies in humans demonstrated that a diet supplemented with passion fruit peel (PFP) flour could improve metabolic parameters, such as reducing fasting glycemia and glycated hemoglobin in type 2 diabetic individuals, as well as reducing fasting glycemia and triglyceride levels in hypercholesteremic women [29]. It was recently found that mamey is a potential source of pro-vitamin A carotenoids (pulp) and dietary fiber (peel).Still, their use as a food ingredient is distant from being commercially available, since there are no reports of actual research because of their small cultivation area [30,31]. In this sense, some commercially available products have been developed from different by-products, but they are not widely accessible worldwide, such as the case of PFP flour developed in Brazil, jackfruit peel and pulp flour in India, lychee seed oil in China, mamey seed oil in several local markets in Mexico, and newly developed açaí seed powder in the United States.

Therefore, the present review analyses the nutritional composition and content of bioactive compounds in lesser-consumed tropical fruits, their by-products (peel and seed), and their antioxidant and anti-inflammatory potentials. The information discussed herein will be useful to understand their possible health benefits. It will serve to increase their consumption, as well as to promote the use of their by-products, which are currently underutilized.

## 2. Methods and Data Collection

Science Direct, Google Scholar, Scopus and Springer databases were used to find information on the composition and bioactivities of lesser-consumed tropical fruits and their by-products. Keywords such as “common and scientific name of the fruit” + peel, pulp, flesh, pomace, seed and by-products, phenolic compounds and bioactivities were used for the data search. Alternatively, the most characteristic bioactive compound present in a fruit were searched with the terms “antioxidant” and “anti-inflammatory”. The data search also included the words “exotic fruits” and “Amazonian fruits” because several sources include these fruits without listing their names in the title or key words.

## 3. Chemical Composition of Peel, Seed, and Pulp of Lesser-Consumed Tropical Fruits

**Figure 1:**
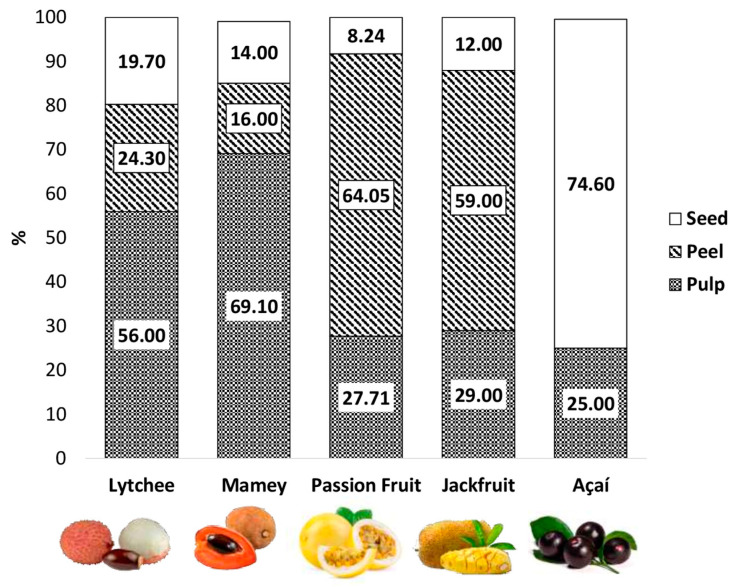
Percentage of pulp, seed, and peel in lesser-consumed tropical fruits (lychee [39], mamey [43], passion fruit [36], jackfruit [41] and açaí [32]).

Figure 1 shows each tropical fruit’s percentage of pulp, peel, and seed, while Table 1 shows their nutritional composition. Açaí seed is approximately 85 to 90% of the fruit’s weight, with its pulp (10%) and peel (2%) comprising the rest. Its pulp contains a high percentage of dietary fiber, carbohydrates, and lipids, while also being a good source of minerals such as calcium, iron, magnesium, and phosphorus. This fruit is also characterized by phenolic compounds, particularly anthocyanins, and some carotenoids, such as α-carotene, β-carotene, lutein, and zeaxanthin (Table 1 and Table 2) [32,33,34,35].

Mamey contains mostly pulp (approximately 70%), with its peel and seed making up a minor percentage of its composition. Its peel is rich in dietary fiber (61.43 g/100 g dw), as compared to the pulp (21.50 g/100 g dw). Its pulp has an energy content of 1287 KJ/100 g dw [31], while the seed is rich in monounsaturated and polyunsaturated fatty acids.

Peel is the major component of passion fruit (approximately 64%), followed by pulp and seed. Its seed is rich in dietary fiber, with a 2:1 ratio of insoluble-to-soluble fiber, followed by protein and lipids. Its fatty acid profile is high in polyunsaturated fatty acids. Its pulp and peel contain mostly carbohydrates, while some of its most representative bioactive compounds include vitamin E and some carotenoids such as lycopene and β-carotene [11,36,37,38].

Lychee seed and peel have similar caloric densities, while the peel is also noteworthy for its high dietary fiber content. Minerals such as calcium, iron, magnesium, phosphorus, and potassium are also found in lychee. Its profile of bioactive compounds is characterized by vitamin C in all tissues, with the highest concentration in the peel. Its content of β-carotene in pulp and peel also stands out with 291.4 and 195.09 µg of β-carotene equivalents/100 g dw, respectively [39,40].

Jackfruit can weigh between 10 and 50 kg when ripe, with most of its weight (59%) concentrated in the peel, followed by pulp and seed [41]. Its pulp contains up to 25% of carbohydrates and low protein content, and it is a good source of minerals such as potassium, magnesium, iron, and calcium [21].

Most lesser-consumed tropical fruits and their by-products are rich in bioactive compounds such as dietary fiber, vitamins, minerals and phenolic antioxidants, and their consumption could have positive health effects that can contribute to prevent some diseases. The addition of these fruits to different dishes, as well as a food ingredient in the development of new food products, is a current trend, for example, in meat products, breakfast cereals, and bakery products, among others [42].

**Table 1:** Nutritional composition of pulp, seed, and peel of mamey, açaí, passion fruit, lychee, and jackfruit.

Component	Mamey	Açaí	Passion Fruit	Lychee	Jackfruit
Water	PP: 61.53 ± 0.42% [43]	PP: 3.4 g/100 g dw [44]	SD: 57.09 g/100 g fw PL: 87.14 g/100 g fw PP: 90.06 g/100 g fw [36]	PL: 68.93 g/100 g PP: 83.91 g/100 g SD: 47.11 g/100 g [45] PP: 80.7% [39]	PP: 72-94 g/100 g fw [46] SD: 51.0-64.5 g/100 g fw [46]
Energy	PP: 1287 ± 26 KJ/100 g dw [31] PL:632 ± 18 KJ/100 g dw [31]	PP: 77 kcal/100 g fw [47]	NR	PP: 70.2 kcal/100 g [39] SD: 397.4 g/100 g [45] PL: 343.04 Kcal/100 g [45]	PP: 88-410 KJ/100 g fw [46] SD: 133–139 KJ/100 g fw [46]
Protein	PP: 4.84 ± 0.07 g/100 g dw [31]	PP: 8.1-21 g/100 g dw [44]	SD:13.07 g/100 g dw PL: 3.40 g/100 g dw PP: 8.57 g/100 g dw [36]	PP: 6.68 g/100 g dw; 0.7% [39,45] SD: 4.83 g/100 g dw [45] PL: 10.86 g/100 g dw [45]	PP: 1.2–1.9 g/100 g fw [46] SD: 20.19% dw [21]
Lipids	PP: 2.82 ± 0.66 g/100 g dw [31]	PP: 32.5-48 g/100 g dw [44]	SD: 12.31 g/100 g dw PL: 4.20 g/100 g dw PP: 1.11 g/100 g dw [36]	PP: 3.80 g/100 g dw; 0.8% [39,45] SD: 2.77 g/100 g dw [45] PL: 6.97 g/100 g dw [45]	PP: 0.1-0.4 g/100 g fw [46] SD: 11.39% dw [21]
Carbohydrates	PL: 65.7 ± 0.4 g/100 g dw [31]	PP: 36 ± 4 g/100 g dw [35]	SD: 71.07 g/100 g dw PL: 85.78 g/100 g dw PL: 83.37 g/100 g dw [36]	PP: 85.38 g/100 g dw; 15.3% [39,45] SD: 86.63 g/100 g dw [45] PL: 85.38 g/100 g dw [45]	PP: 16-25.4 g/100 g fw [46] SD: 25.8-38.4 g/100 g fw [46] SD: 51.82% dw [21]
Total dietary fiber	PP: 21.50 ± 1.13 dw [48] PP: 22.29 g/100 g dw [31] PL: 61.43 g/100 g dw [31]	PP: 44.2 g/100 g dw [44]	SD: 65.60 g/100 g dw PL: 61.16 g/100 g dw PP: 7.15 g/100 g dw [36]	PP: 2.47 g/100 g; 2.2% [39,45] SD: 4.07 g/100 g dw [45] PL: 18.21 g/100 g dw [45]	SD: 7.10% dw [21]
Total sugars	PP: 55.81 ± 0.39 [43]	NR	NR	NR	NR
Calcium	NR	PP: 260 mg/100 g dw [44]	SD:0.030 mg/100 g PL: 0.25 mg/100 g PP: 0.05 mg/100 g [36]	PP: 1.80 mg/100 g dw [39]	SD: 190 ppm dw [21]
Iron	PP: 0.0052–0.0262 g/kg [49]	PP: 49.8 mg/kg dw [50]	SD:0.0052 mg/100 g PL: 3.20 mg/100 g PP: 0.0055 mg/100 g [36]	PP: 0.8 mg/100 g [39]	SD: 148.5 ppm dw [21]
Magnesium	PP: 0.28–1.21 g/kg [49]	PP: 286 mg/kg dw [50]	SD: 0.094 mg/100 g PL: 0.12 mg/100 g PP: 0.02 mg/100 g [36]	PP: 12.90 mg/100 g [39]	SD: 240 ppm dw [21]
Phosphorus	PP: 0.28–0.30 g/kg [49]	PP: 186 ± 1.5 mg/100 g dw [35]	PL: 0.310 mg/100 g [36]	NR	
Potasium	PP: 2.26 g/kg [49]	PP: 930 ± 9.9 mg dw [35]	SD: 0.760 mg/100 g PL: 2.60 mg/100 g PP: 3.8 mg/100 g [36]	PP: 1067.33 mg/100 g [39]	SD: 2470.00 ppm dw [21]
Sodium	PP: 0.06–0.10 g/kg [49]	PP: 6.8 ± 0.7 mg/100 g dw [35]	SD: 0.0041 mg/100 g PL: 0.0022 mg/100 g PP: 0.0014 mg/100 g [36]	PP: 5.9 mg/100 g [39]	SD: 398.50 ppm dw [21]
Zinc	NR	PP: 2.1 mg/100 g dw [35]	SD: 0.0041 g/100 g PL: 1.00 mg/100 g [36] PP: 1.9 mg/100 g [23]	PP: 0.22 mg/100 g [39]	SD: 40.85 ppm dw [21]
Vitamin C	PP: 29.37 ± 3.58 mg of vitamin C/100 g fw [48]	PP: <0.1 mg/100 g dw [44]	NR	PP: 26.9 mg/100 g [39]	PP: 7.0-10.0 mg/100 g fw SD: 11 mg/100 g fw [46]
Total saturated fatty acids	SD: 39.91 g/100 g [43]	NR	SD: 14.69 g/100 g [23]	NR	NR
Total mono unsaturated fatty acids	SD: 48.62 g/100 g [43]	NR	SD: 17.18 g/100 g [23]	NR	NR
Total polyunsaturated fatty acids	SD: 11.35 g/100 g [43]	NR	SD: 68.12 g/100 g [23]	NR	NR

SD: seed; PP: pulp; PL: peel; AAE: ascorbic acid equivalents; dw: dry weight; fw: fresh weight; NR: not reported. Units are shown unmodified from the original sources.

## 4. Phytochemical Content

Phytochemicals are secondary plant metabolites that protect plant tissues against various biotic and abiotic stresses. Some of them play important roles in human health, for example, edible plants contain phenolic compounds, carotenoids, and tocopherols, which are associated with beneficial effects on the prevention of cardiovascular disease risk factors, inhibition of inflammation, reducing oxidative stress and preventing or delaying oxidation by scavenging free radicals [51,52]. Recognizing the presence of phytochemicals in lesser-consumed tropical fruits and their by-products requires knowing their quantities and diversity to investigate their possible effects on human health, and this is described in this section. Table 2 describes the content of several bioactive compounds present in lesser-consumed tropical fruits and their by-products. It should be noted that flavonoids are the predominant phenolic species present in these fruits and the ones that this review focuses on the most; however, other minor components may also be present (such as non-flavonoids), but have been less studied in these fruits.

### 4.1. Phenolic Compounds

Phenolic compounds have a common basic structure with significant diversity, which is precisely why their physicochemical properties are diverse. Complex glycosylation and polymerization patterns complicate their extraction, purification, and identification and, therefore, different methods are required for these purposes [53]. Table 3 shows the content of phenolic compounds found in lesser-consumed tropical fruits and their by-products, as obtained by different extraction methods.

Jackfruit peel has the highest phenolic content (4804 mg gallic acid equivalents (GAE)/100 g dw), which is nearly fivefold higher than passion fruit peel (1061.87 mg GAE/100 g dw) [22,36,40]. Açaí seed can contain up to 49,099 mg GAE/100 g dw, followed by jackfruit (971 GAE/100 g dw), passion fruit (346.69 mg GAE/100 g dw) and lychee (34.72 mg GAE/100 g dw) [22,36,40,54].

Açaí pulp also has a high phenolic content, but it has been observed that its highest concentration is found in unripe fruits (12,317 mg GAE/100 g dw), which gradually decreases until fully ripe (3437 mg GAE/100 g dw). It has been observed that, in general, unripe fruit had the highest phenolic content. Passion fruit (1297.31 mg GAE/100 g dw) and jackfruit pulp (1034–1157 mg GAE/100 g dw) contain lower concentrations, followed by lychee (20.30 mg GAE/100 g dw) [22,26,36]. Phenolic compounds in mamey have also been reported to vary by up to 10-fold from unripe (256.3 mg GAE/100 g fw) to ripe (23.4 mg GAE/100 g fw) fruit, while senescent mamey fruit shows further decreases (6.6 mg GAE/100 g dw) [55,56].

#### 4.1.1. Phenolic Acids

The highest concentration of phenolic acids is found in mamey and the lowest in lychee, with the most representative compounds being *p*-hydroxybenzoic acid in mamey (484 mg/100 g dw), gallic acid in açaí (6.87 mg/100 g dw) and 5-caffeoylquinic acid in jackfruit (3.42 mg/100 g dw) [10,11,20,56,57,58,59,60,61].

#### 4.1.2. Flavonoids

Flavonoids are subclassified as flavanols, flavonols, flavanones, flavones, flavonones and anthocyanins. Total flavonoid concentration in lesser-consumed fruits has been reported as 87,140 mg of quercetin equivalents (QE)/100 g dw, 227 mg QE/100 g dw and 162 mg QE/100 g dw in jackfruit peel, pulp, and seed, respectively. Some specific flavonoids have been reported, such as 158.037 mg of rutin equivalents (RE)/L fw in passion fruit pulp, 65.24 mg QE/100 g fw in mamey pulp, and 7.0 mg RE/100 g fw in açaí pulp [10,28,48,62].

##### Flavanols

Catechin and epicatechin can be found in mamey, açaí and lychee pulps as representative compounds. Reported values of catechin in mamey and lychee are 11.31 and 0.486 mg/100 g fw, respectively. In açaí pulp, catechin values of 5.07 mg/100 g dw have been reported. For epicatechin, values of 0.58 mg/100 g fw in mamey pulp and 0.498 mg/100 g fw in lychee pulp have been reported. In açaí pulp, 2.09 mg/100 g dw of epicatechin was reported. Gallocatechin-3-gallate is found in mamey and açaí pulp, while gallocatechin and catechin-3-*O*-gallate is found in mamey pulp. A total flavanol content of 50.65 mg/100 g dw have been reported in açaí pulp [56,57,58,60,63,64].

##### Flavonols

Flavonols are found in most of these less-consumed fruits, except for jackfruit, and are significant in açaí and lychee. For example, rutin (3.89 mg/100 g dw), quercetin (13,566 mg/100 g dw), isorhamnetin rutinoside (1.7 mg/100 g dw) and kaempferol (0.521 mg/100 g dw) are found in açaí pulp, along with others. In mamey pulp, dihydromyricetin (200.77 ppm fw) was reported, as well as myricitrin (25.48 ppm fw) [10,59,60,63].

##### Flavones

Flavones have been identified in açaí pulp, where orientin and isovitexin stand out, with 15.0 and 12.0 mg/100 g dw, respectively. Passion fruit peel also contains isoorientin, with 19.63 mg/100 g dw and its pulp has 16.226 mg/L fw [10,11,62]. This phenolic species is widely spread in the plant kingdom and has been reported in several fruits, vegetables, cereals, legumes, and wines [65]. Nevertheless, no studies have been addressed to identify and quantify them in lesser-consumed tropical fruits such as lychee, mamey, jackfruit or their by-products.

##### Anthocyanins

Açai pulp is characterized by its abundant anthocyanin content (587 mg cyanidin-3-glucoside equivalents/100 g dw), although significant variation is common. This is attributed to the fruit’s high perishability and anthocyanins’ susceptibility to degradation, different extraction, and quantification methods, in addition to seasonal, geographic and ripeness variation [66]. Pelargonidin-3-glucoside and cyanidin-3-glucoside are among the most representative compounds, with 111.92 and 67.33 mg/100 g dw, respectively [17,60,67,68]. While mamey and jackfruit contain 5.57 and 0.46 mg of total anthocyanins (TA)/100 g fw, respectively [48,69].

### 4.2. Carotenoids

Carotenoids are an important and widespread type of phytochemicals found in plants and plant-derived food, bacteria, fungi, and animals, with several health-promoting properties attributed to them. They are tetraterpene pigments, of which more than 700 have been identified in nature [70,71]. Carotenoids are commonly found in lesser-consumed tropical fruits and their by-products.

Carotenoid content in mamey, passion fruit and jackfruit have been reported as 36.12 mg β-carotene/100 g fw, 25.10 mg/100 g fw and 0.592 mg/100 g fw, respectively. In açaí, concentrations of 4.23 mg/100 g dw have been reported [17,41,48,72]. Açaí contains lutein, α-carotene, 13-*cis*-β-carotene, and 9-*cis*-β-carotene [17,67]. Jackfruit pulp contains *trans*-lutein (24–44%), *trans*-β-carotene (24–30%), *trans*-neoxanthine (4–19%), 9-*cis*-neoxanthine (4–9%), and 9-*cis*-violaxanthin (4–10%) [6,73].

Mamey is distinguished for its carotenoid composition, since sixty-two carotenoids and carotenoid esters in saponified and non-saponified mamey pulp extracts have been identified. Likewise, twenty-three compounds that belong to seventeen different chemical classes of carotenoids have been identified. The most representative molecules include neoxanthin, cryptocapsin, luteoxanthin and capsoneoxanthin [19,70].

Mamey is also notable for its carotenoid content with a kappa terminal group, which are not very common; the kappa ring is usually hydroxylated, such as in capsanthin, capsorubin, and cryptocapsin. Studies suggest that mamey contains two enzymes that carry out the biosynthesis of kappa-carotenoids, one that catalyzes the epoxidation of the non-hydroxylated β-ring and another that reorganizes the epoxides [30,74]. As noted, lesser-consumed tropical fruits are a significant source of carotenoids, which is of interest because of the bioactivities these compounds can perform. Evidences indicate that these phytochemicals contribute to the decrease in the incidence of diseases and protect against the recurrence of pathological events [63,71].

**Table 2:** Phytochemical content in the pulp, seed, and peel of mamey, açaí, passion fruit, lychee, and jackfruit.

Phenolic Acids	
Phytochemical	Content
Gallic acid	Mamey PP: 0.47 mg/100 g fw [56]; 1.92 mg/100 g dw [57]; 170.91 ± 0.53 ppm fw [63] Açaí PP:6.87 ± 0.28 mg/100 g dw [60] Lychee: cv Qingke: 0.1055, cv Baila: 0.063, cv Jizui: 0.048 mg/100 g fw [58]
p-hydroxybenzoic acid	Mamey PP: 484 mg/100 g dw [63] Açaí PP: 1.0 ± 0.8 mg/100 g dw [10] Passion fruit: 0.0124 ± 0.0011 mg/100 g fw [11] Jackfruit PP: 19.978 ± 1.66 mg/g dw [75]
Protocatechuic acid	Açaí PP: 0.717 ± 0.054 mg/100 g [59]; PP: 1.7 ± 0.4 mg/100 g dw [10]
Protocatechuic acid hexoside	PP: 0.9 ± 0.6 mg/100 g dw [10]
Chlorogenic acid	Açaí PP: 0.909 ± 0.102 mg/100 g [59]; PP: 5.01 ± 0.78 mg/100 g dw [60] Passion fruit: 0.0183 ± 0.002 mg/100 g fw [11] Lychee: cv Qingke:0.008, cv Baila: 0.0219, cv Jizui: 0.064 mg/100 g fw [58]
Caffeic acid	Açaí PP: 0.238 ± 0.018 mg/100 g [59]; PP: 0.61 ± 0.22 mg/100 g dw [60]; PP: 1.9 ± 0.8 mg/100 g dw [10] Passion fruit: 0.0056 ± 0.0005 mg/100 g fw [11] Lychee: cv Qingke: 0.0621, cv Baila: 0.0576, cv Jizui: 0.114 mg/100 g fw [58]
Vanillic acid	Açaí PP: 4.655 ± 0.233 mg/100 g [59]; PP: 11.0 ± 5.8 mg/100 g dw [10] Passion fruit: 0.0426 ± 0.0029 mg/100 g fw [11]
Syringic acid	Açaí PP: 1.903 ± 0.120 mg/100 g [59]; PP: 1.62 ± 0.37 mg/100 g dw [60]; PP: 4.8 ± 1.1 mg/100 g dw [10] Lychee PP: 3.96 ± 0.95 μg/g fw [76]
Synapic acid	Açaí PP: 0.082 ± 0.010 mg/100 g [59]
*p*-coumaric acid	Açaí PP: 0.22 ± 0.015 mg/100 g [59]; PP: 1.74 ± 0.33 mg/100 g dw [60] Passion fruit: 0.024 ± 0.0015 mg/100 g fw [11] Lychee PP: 0.894 ± 0.119 mg/g dw [76]
Ferulic acid	Açaí PP: 0.322 ± 0.020 mg/100 g [59] Passion fruit: 0.0015 ± 0.0003 mg/100 g fw [11] Lychee PP: 6.26 ± 1.01 μg/g fw [76]
Kaftaric acid	Açaí PP: 0.86 ± 0.10 mg/100 g dw [60]
5-caffeoylquinic acid	Açaí PP: 4.3 mg/100 g dw [10] Passion fruit PP: 0.0104 mg/100 g [61] Jackfruit PP: 3.42 ± 0.04 mg/100 g [20]
4-caffeoylquinic acid	Passion fruit PP: 0.012 mg/100 g [61] Jackfruit 0.144 ± 0.004 mg/100 g [20]
3,5-dicaffeoylquinic acid	Passion fruit PP: 0.0576 mg/100 g [61] Jackfruit PP: 0.131 ± 0.01 mg/100 g [20]
4,5-dicaffeoylquinic Acid	Passion fruit PP: 0.0587 mg/100 g [61] Jackfruit PP: 0.050 ± 0.004 mg/100 g [20]
*p*-coumaric acid, hexoside	Açaí PP: 1.0 ± 0.5 mg/100 g dw [10]
Isomer 1 of feruloyl sinapic acid	Açaí PP: 1.3 ± 0.6 mg/100 g dw [10]
Feruroylhydroxypyruvic acid	Açaí PP: 1.4 ± 0.5 mg/100 g dw [10]
Isomer 1 of caffeoyl shikimic acid	Açaí PP: 1.7 ± 1.5 mg/100 g dw [10]
Isomer 2 of feruloyl sinapic acid	Açaí PP: 0.8 ± 0.3 mg/100 g dw [10]
Isomer 2 of caffeoyl shikimic acid	Açaí PP: 5.4 mg/100 g dw [10]
Sinapoyl hexose	Açaí PP: 1.0 ± 0.8 mg/100 g dw [10]
Feruloylquinic hydroxy acid	Açaí PP: 0.7 ± 0.4 mg/100 g dw [10]
Sinapoyl rhamnose	Açaí PP: 1.4 ± 0.9 mg/100 g dw [10]
Feruloyl derivative	Açaí PP: 2.3 ± 0.7 mg/100 g dw [10]
Flavanols	
Catechin	Mamey PP: 0.99 -11.31 mg/100 g fw [56]; 75.01 ± 2.67 ppm fw [63] Açaí PP: 5.07 ± 0.48 mg/100 g dw [60] Lychee cv Qingke: 0.486, cv Baila: 0.246, cv Jizui: 0.215 mg/100 g fw [58]
Galocatechin-3-gallate	Mamey PP: 1.19 mg/100 g fw [56] Açaí PP: 25.00 ± 0.64 mg/100 g dw [60]
Gallocatechin	Mamey PP: 172.85 ± 2.21 ppm fw [63] Lychee PP: 2307.91 ± 66.76 μg/g fw [76]
Catechin-3-*O*-gallate	Mamey PP: 80.50 ± 0.81 ppm fw [63]
Epicatechin	Mamey PP: 0.58 mg/100 g fw [56];0.78 mg/100 g dw [57]; 24.42 ± 0.97 ppm fw [63] Açaí PP: 2.03 ± 0.09 mg/100 g dw [60] Lychee cv Qingke: 0.498, cv Baila: 0.393, cv Jizui: 0.249 mg/100 g fw [58]; PP: cv Hemaoil: 0.0425, cv Feizixiao: 0.0196, cv Lanzuhu: 0.008 mg/100 g dw [64]
Flavonols	
Flavonoids	Mamey PP: 65.24 ± 4.49 mg quercetin/100 g fw [48] Passion fruit PP: 158.037 ± 0.602 mg/L fw [62] Jackfruit PL: 279 ± 4; PP: 227 ± 31; SD: 162 ± 10 mg quercetin/100 g dw [22]; PL: 87,140 mg QE/100 g dw [28]
Rutin	Açaí PP: 3.89 ± 0.15 mg/100 g dw [60]; PP: 3.4 ± 0.7 mg/100 g dw [10] Passion fruit PP: 0.0227 ± 0.0027 mg/100 g fw [11] Lychee cv Qingke: 0.591, cv Baila: 0.563, cv Jizui: 1.888 mg/100 g [58]; PP: cv Hemaoil: 0.009, cv Feizixiao: 0.065, cv Lanzuhu: 0.023 mg/100 g dw [20]
Isorhamnetin rutinoside	Açaí PP: 1.7 ± 0.3 mg/100 g dw [10]
Dihydromyricetin	Mamey PP: 200.77 ± 11.73 ppm fw [63]
Myricitrin	Mamey PP: 25.48 ± 3.70 ppm fw [63]
Quercetin	Açaí PP: 13.566 ± 0.098 mg/100 g dw [59] Passion fruit PP: 0.0416 ± 0.0006 mg/100 g fw [11] Lychee PP: 1.325 ± 0.007 mg/g dw [76]
Quercetin-3-glucoside	Açaí PP: 1.54 ± 0.34 mg/100 g dw [60]
Kaempferol	Açaí PP: 0.521 ± 0.036 mg/100 g dw [59]
Flavanones	
Naringenin	Açaí PP: 1.64 ± 0.48 mg/100 g dw [60]
Hesperidin	Açaí PP: 1.96 ± 0.51 mg/100 g dw [60]
Flavones	
Isovitexin	Açaí PP: 12.0 ± 4.8 mg/100 g dw [10] Passion fruit PP: 2.76 mg/100 g dw [20]
Homoorientine	Açaí PP: 9.9 ± 4.9 mg/100 g dw [10]
Vitexin	Açaí PP: 9.8 ± 5.2 mg/100 g dw [10]
Escoparina	Açaí PP: 0.6 ± 0.2 mg/100 g dw [10]
Chrysoeriol	Açaí PP: 0.5 ± 0.3 mg/100 g dw [10]
Orientin	Açaí PP: 15.0 ± 6.3 mg/100 g dw [10] Passion fruit PL: 0.970 mg/100 g dw [20]
Isoorientin	Passion fruit PL: 19.63 mg/100 g dw [62]; PP: 16.226 ± 0.050 mg/L fw [11]
Luteolin	Açaí PP: 2.161 ± 0.216 mg/100 g [59]; PP: 0.9 ± 0.3 mg/100 g dw [10]
Apigenin	Açaí PP: 1.257 ± 0.134 mg/100 g [59]
Flavonones	
Taxifolin deoxyhexose isomer 1	Açaí PP: 2.8 ± 1.7 mg/100 g dw [10]
Taxifolin deoxyhexose isomer 2	Açaí PP: 1.3 ± 0.7 mg/100 g dw [10]
Taxifolin	Açaí PP: 1.2 ± 0.4 mg/100 g dw [10]
Anthocyanins	
Malvidin-3-glucoside	Açaí PP: 6.9 ± 0.82 mg/100 g dw [60]
Malvidin-3.5-diglucoside	Açaí PP: 11.51 ± 1.37 mg/100 g dw [60]
Cyanidin-3-glucoside	Açaí PP: 67.33 ± 1.06 mg/100 g dw [60]; PP: 0.13–541.5 mg/100 g fw [44]
Cyanidin-3-rutinoside	Açaí PP: 2.57–1395.3 mg RAE/100 g fw [44]
Pelargonidin-3-glucoside	Açaí PP: 111.92 ± 3.04 mg/100 g dw [60]
Peonidin-3-glucoside	Açaí PP: 1.32 ± 0.29 mg/100 g dw [60]
Total anthocyanins	Mamey: PP: 5.57 ± 0.07 mg TA/100 g fw [48] Açaí PP: 35.41 mg of cianidine-3-glucoside equivalent/100 g fw [68]; PP: 587 ± 53 mg cyanidin-3-glucoside equivalents/100 g of dw [67] Jackfruit PP: 0.46 mg TA/100 g fw [69]
Proanthocyanidins	
Procyanidin B1	Açaí PP: 1.99 ± 0.36 mg/100 g dw [60]
Procyanidin B2	Açaí PP: 5.03 ± 0.4 mg/100 g dw [60] Lychee PP: cv Hemaoil: 39.93, cv Feizixiao: 0.032, cv Lanzuhu: 0.017 mg/100 g dw [20]
Procyanidin A2	Açaí PP: 11.53 ± 1.53 mg/100 g dw [60] Lychee PP: cv Hemaoi: 0.018, cv Feizixiao: 0.001 mg/100 g dw [20]
Stilbenes	
*trans*-resveratrol	Açaí PP: 0.38±0.14 mg/100 g dw [60]
Carotenoids	
Neoxanthin	Mamey PP: Genotype 8747: 1.024 ± 0.263, Genotype 11,129: 0.370 ± 0.099 mg/100 g dw [19] Jackfruit PP: All-*trans*-neoxanthin: 8.85 μg/100 g wm; 9-*cis*-neoxanthin: 6.87 μg/100 g [73]
Lycopene	Lychee SD: 0.0043 mg/100 g [77]
Violaxanthin	Mamey PP: Genotype 8747: 0.360 ± 0.119, Genotype 11,129: 0.164 ± 0.057 mg/100 g dw [19]
Luteoxanthin	Mamey PP: Genotype 8747: 0.569 ± 0.163, Genotype: 11,129: 0.180 ± 0.0 80 mg/100 g dw [19]
Lutein and zeaxanthin	Açaí PP: 0.367 ± 0.142 mg/100 g dw [17]; PP: 0.717 mg/100 g dw [44] Passion fruit Lutein; PL: 0.504 mg/100 g dw [36]; Zeaxanthin; PL: 0.065 mg/100 g dw [36]; PP: 0.044 mg/100 g dw [36] Passion fruit PP: All-*trans*-lutein: 37.02 μg/100 g fw; All-*trans*-zeaxanthin: 0.96 μg/100 g fw [75]
Capsoneoxanthin	Mamey PP: Genotype: 8747: 1.428 ± 0.402, Genotype: 11,129: 0.454 ± 0.170 mg/100 g dw [19]
α-Carotene	Açaí PP: 0.450 ± 0.002 mg/100 g dw [17] Jackfruit PP: All *trans*-αcarotene: 1.24 μg/100 g fw [73]
β-cryptoxanthin epoxide	Mamey PP: Genotype 8747: 0.208 ± 0.058, Genotype 11,129: 0.042 ± 0.020 mg/100 g dw [19] Passion fruit PL: 0.075 mg/100 g dw [36]; PP: 0.254 μm/100 g dw [36] Jackfruit PP: 1.21 μg/100 g fw [73]
13-*cis*-β-Carotene	Açaí PP: 0.055 ± 0.037 mg/100 g dw [17] Jackfruit PP: 2.45 μg/100 g fw [73]
9-*cis*-β-Carotene	Açaí PP: 0.365 ± 0.002 mg/100 g dw [17] Jackfruit PP: 0.79 μg/100 g fw [73]
β-Carotene	Mamey 1.2–1.5 mg β-carotene/100 g [78] Açaí PP: 0.010–0.149 mg/100 g dw [44] Passion fruit PL: 0.272; PP: 1.334 mg of β-carotene equivalents mg/100 g dw [36] Lychee SD: 2.77 mg/mL [77]; PL: 195.09 mg/mL [39]; PP: 0.291 mg of β-carotene equivalents/100 g fw [39] Jackfruit PP: All *trans*-β-carotene 29.55 μg/100 g fw [73]
Total carotenoids	Mamey PP: Genotype 8747: 8.076, Genotype 11,129: 3.786 mg/100 g fw [19]; PP: 36.12 ± 1.24 mg β-carotene/100 g fw [48]; PP: 1.127 ± 0.005 mg β-carotene/100 g fw [57] Açaí PP: 4.15 ± 0.41 mg/100 g dw [67]; PP: 4.2345 ± 0.007 mg/100 g dw [17] Passion fruit PP: 25.10 mg/100 g fw [72] Jackfruit PP: 0.592 mg/100 g fw [41]; PP: 107.98 μg/100 g fw [73]
Tocopherols	
Total tocopherols	Passion fruit PP: 0.52 mg/100 g fw [72]
δ- Tocopherol	Mamey PP: 0.360 ± 0.030 mg/100 g dw [57]
Ascorbic acid	
Vitamin C	Mamey PP: 29.37 ± 3.58 mg vitamin C/100 g fw [48] Lychee PP: 34.7 ± 7.8 mg vitamin C/100 g fw [39]

dw: dry weight; fw: fresh weight; PP: pulp; PL: peel; SD: seed; TA: total anthocyanins. Units are shown unmodified from the original sources.

## 5. Bioactivities

### 5.1. Antioxidant Activity (AOXA)

As can be noticed in Table 3, lesser-consumed tropical fruits have shown significant AOXA. The AOXA of jackfruit, as determined with the 2,2′-azino-bis(3-ethylbenzothiazoline-6-sulfonic acid) (ABTS^•^) assay, is reported as IC_50_ = 570, 23 and 762 mg dw/100 mL extract in pulp, peel, and seed, respectively. The results of DPPH^•^ are reported as 16 μM Trolox equivalents (TE)/100 g fw, 1.25 mg dw/mL and >10 mg dw/mL in pulp, peel, and seed, respectively. In the same way, an in vitro assay of α-glucosidase inhibition was developed and indicated that peel extract inhibited about 11.8-fold, as compared to acarbose [22,69]. AOXA values have been reported to positively correlate with the total phenolic compounds content in jackfruit when using the ABTS^•^ (R = 0.94, *p* ≤ 0.001) and DPPH^•^ (R = 0.88, *p*≤ 0.001) assays, higher values than the correlation with ascorbic acid (R = 0.38 and R = 0.50, respectively) and anthocyanins (R = 0.36 and R = 0.19, respectively), suggesting that phenolic compounds other than anthocyanins, such as phenolic acids, tannic acid and proanthocyanidins, may be the most important contributors to the AOXA of this fruit [69]. Therefore, the results reveal the potential of jackfruit peel as a source of natural antioxidants and hypoglycemic agents.

In vitro assays have shown that extracts from jackfruit pulp have ABTS^•^ radical scavenging capacity, with an inhibition of 11.7%. Jackfruit extracts have also shown a greater capacity to inhibit nitric oxide (NO^•^) (75.3% inhibition) and superoxide anion (O^•−^) (46% inhibition) than other fruits that are commonly known as good inhibitors of NO^•^ and O_2_^•−^, such as blueberries (44.2 and 10%), black raspberry (13.1 and 8.7%), grapes (66.9 and 43%) and red raspberry (37 and 43%). The capacity of jackfruit pulp extracts to stabilize DPPH^•^, ABTS^•^, NO^•^ and O_2_^•−^ has shown positive correlation with total phenolic content (R = 0.967, 0.621, 0.380 and 0.532), flavonoids (R = 0.30, 0.995, 0.685 and 0.802) and proanthocyanidins (R = 0.902, 0.755, 0.201 and 0.371). This suggests that the stabilization of the DPPH^•^ radical is mainly due to the presence of total phenolic compounds, including proanthocyanidins and the stabilization of the ABTS^•^ radical is due to the presence of the flavonoids and proanthocyanidins, while the inhibition of NO^.^ and O_2_^•−^ is due to the presence of flavonoids [79].

AOXA of mamey has been reported as 393.81 and 113.06 μmol TE/100 g fw for ABTS^•^ and DPPH^•^, respectively, and these values are mainly attributed to the soluble phenolic compounds. Furthermore, AOXA by the DPPH^•^ method shows that catechin-3-*O*-gallate and gallocatechin, both present in mamey, had an IC_50_ = 19.0 and 20.7 μM, respectively [48,63].

AOXA of açaí pulp has been reported as 5795 and 93,682 μM Trolox/100 g dw according to the ABTS^•^ and DPPH^•^ assays, respectively. Fruit ripening has been shown to directly influence AOXA of açaí, where AOXA of unripe (green), intermediate maturity (reddish-brown) and ripe (dark purple) has been reported as 17.0, 4.04 and 2.78 μM TE/100 g dw, respectively [35].

DPPH^•^ radical scavenging capacity of passion fruit shows that seed extracts had the lowest IC_50_ value (49.71 µg/mL), indicating a stronger AOXA than in the peel and pulp, which had values of 347.6 and 869.05 µg/mL, respectively. According to the ferric reducing antioxidant power (FRAP) assay, AOXA varied between 27.50 and 119.32 µM of FeSO_4_/g, with seed extracts showing the highest values [11].

Polyphenolic compounds have been intensely studied for their anti-inflammatory potential during the past century, since during severe inflammation, cells produce several pro-inflammatory reactive oxygen species (ROS), such as singlet oxygen and NO, while also activating cyclooxygenases (COX) that produce additional molecules [80,81,82]. The anti-inflammatory activities of lesser-consumed tropical fruits and their by-products are discussed in the following section.

**Table 3:** Total phenolic compounds present in the pulp, seed, and peel of mamey, açaí, passion fruit, lychee and jackfruit, and their antioxidant activity.

Sample	Extraction Solvent	Solid: Liquid Ratio	Method	Total Phenolic Compounds (mg GAE/100 g dw)	Antioxidant Activity	Reference
Jackfruit (peel, pulp, and seed)	90% methanol	1:30	6 h stir at 100 rpm	PL: 4804 ± 457; PP:1034 ± 16; Flake: 1157 ± 6 SD: 971 ± 6	IC_50_ mg dw/mL DPPH: PL: 1.25 ± 0.14; PP > 10; SD > 10 ABTS PL: 0.23 ± 0.02; PP: 5.70 ± 0.37; SD: 7.62 ± 0.13	[22]
Jackfruit pulp	60% methanol 0.1% HCl	5:10 (*w*/*v*)	Water bath for 2 h at 85 °C	29.0 ± 6.3 fw	ABTS 0.63 ± 0.0; DPPH 0.16 ± 0.03 μM TE/g fw	[69]
Passion fruit seed	Ethanol	1:4 (*w*/*v*)	Homogenized by exhaustive extraction	346.69	DPPH: IC_50_ = 1.18 ± 0.03 g/100 mL ABTS: IC_50_ = 3.84 ± 0.08 g/100 mL	[36]
Passion fruit seed	Ethanol:water	1/10 (*w*/*v*)	Thermostatic bath under constant agitation	3.11	DPPH IC_50_ = 26.96 ± 0.34 μg/mL FRAP: 3.6 ± 0.29 μg AAE/g ORAC: 6.2 ± 0.53 μmol TE/g	[23]
Lychee seed	Methanol: water (50:50 *v*/*v*)	NR	3 consecutive refluxes at 80 °C	11.45 wm 34.72	NR	[40]
Lychee seed	Ethanol:water (50:50 *v*/*v*)	1:30 (*w*/*v*)	Heating to 50 °C, in a water bath with intermittent mixing at 200 rpm for 50 min	12.90 wm	TEAC: 21.40 ± 1.98 μmol Trolox/g	[26]
Açaí seed extract	Ethanol	1:2 (*w*/*v*)	Boiled in 400 mL of water. 400 mL of ethanol was added. Stirred 2 h a day for 10 days	26,500	NR	[83]
Açaí seed	Ethanol/water	57/43 (*v*/*v*) 1:10 (*w*/*v*)	10 g mixed with 100 mL of ethanol/water (57/43, *v*/*v*), sonicated for 15 min and centrifuged at 5000× *g*	49,099 ± 8	NR	[54]
Passion fruit peel	Ethanol	1:4 (*w*/*v*)	Homogenized by exhaustive extraction	1061.87	DPPH: IC_50_ = 1.69 ± 0.03 g/100 mL ABTS: IC_50_ = 2.22 ± 0.01 g/100 mL	[36]
Passion fruit peel	Water/ethanol/formic acid (94/5/1; *v*/*v*/*v*)	1:4 (*w*/*v*)	Extraction with pressurized hot water. 2.5 g sample, 99 °C (at 50 bar), 7 min extraction	2496	DPPH: 718.91 ± 40.55 μg/mL TEAC: 0.08 ± 0.01 mmol Trolox/g	[84]
Lychee peel	Methanol:water (50:50 *v*/*v*)	NR	3 consecutive refluxes at 80 °C	22.04 fw 71.71	NR	[40]
Lychee peel	Ethanol:water (50:50 *v*/*v*)	1:30 (*w*/*v*)	Heating to 50 °C, in a water bath with intermittent mixing at 200 rpm for 50 min.	25.10	TEAC: 43.80 ± 2.02 μmol Trolox/g	[26]
Passion fruit pulp	Ethanol	1:4 (*w*/*v*)	Homogenized by exhaustive extraction	1297.31	DPPH: IC_50_ = 0.20 ± 0.03 g/100 mL ABTS: IC_50_ = 0.82 ± 0.03 g/100 mL	[36]
Lychee pulp	Methanol: water (50:50 *v*/*v*)	NR	3 consecutive refluxes at 80 °C	21.20 fw	NR	[40]
Lychee pulp	Ethanol:water (50:50 *v*/*v*)	1:30 (*w*/*v*)	Heating to 50 °C with intermittent mixing at 200 rpm for 50 min	20.30	TEAC: 13.20 ± 1.52 μmol Trolox/g	[26]
Açaí pulp	CO_2_	5 g pulp	50 °C/350 bar, 60 °C/420 bar, and 70 °C/490 bar. Solvent mass flow rate of 8.85 ×10^−5^ kg/s and 0.005 kg of dry matter	1542.82	TEAC: 5795 μM Trolox/100 g dw DPPH: 93,682 μM Trolox/100 g dw	[85]
Açaí pulp	Methanol	1:2 *w*/*v*	Sonicated with methanol for 20 min/16 °C, centrifuged at 2800× *g*/10 min. Pellet re-extracted with methanol/water (80:20, *v*/*v*) until discoloration.	4786 ± 1880	ABTS: 24.7 ± 10.6 μmol TE/100 g dw DPPH: 21,049 ± 3071 μmol TE/100 g dw	[10]
Açaí pulp	Not mentioned	50 g pulp	High pressure 600 MPa/5 min/25ºC	235.70	FRAP: 31.3 μmol TE/g ORAC: 42.7 μmol TE/g	[68]

PL: peel; PP: pulp; SD: seed; dw: dry weight; fw: wet weight; rpm: revolutions per minute; h: hours; AAE: ascorbic acid equivalents; TE: Trolox equivalents; NR: not reported. Units are shown unmodified from the original sources.

### 5.2. Anti-Inflammatory Activity

**Figure 2:**
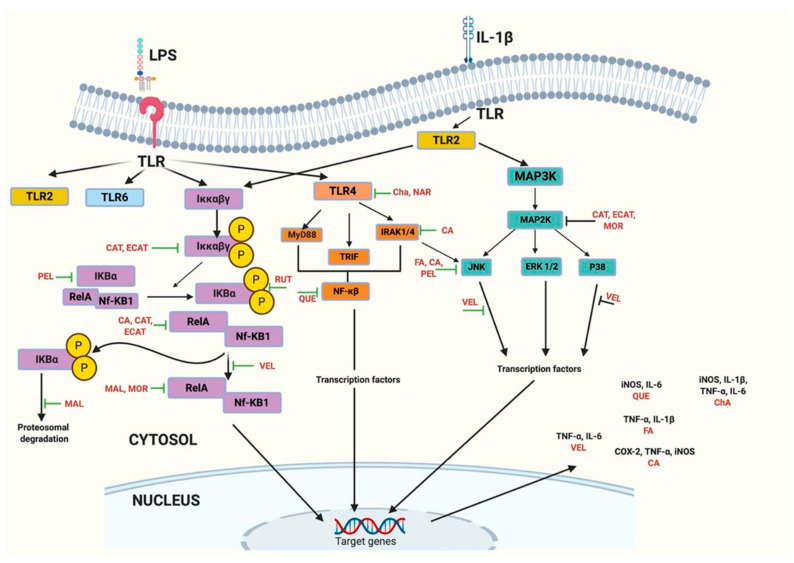
Mechanism of action of some phenolic compounds found in lesser-consumed tropical fruits (lychee, mamey, passion fruit, açaí and jackfruit) and their ability to regulate the inflammatory process. Phenolic compounds can inhibit pro-inflammatory mediators such as IL-6, iNOS, IL-1β, TNF-α, COX-1, COX-2 by inhibiting their activity or gene expression. In addition, some phenolic compounds can up/downregulate transcriptional factors, such as nuclear factor-κB (NF-κB) or Nrf-2, in inflammatory and antioxidant pathways. PEL: pelargonidine; CAT: catechin; ECAT: epicatechin; CA: caffeic acid; MOR: moracin; MAL: malvidin; VEL: velutin; RUT: rutin; QUE: quercetin; Cha: chlorogenic acid; NAR: naringenin; FA: ferulic acid.

**Figure 3:**
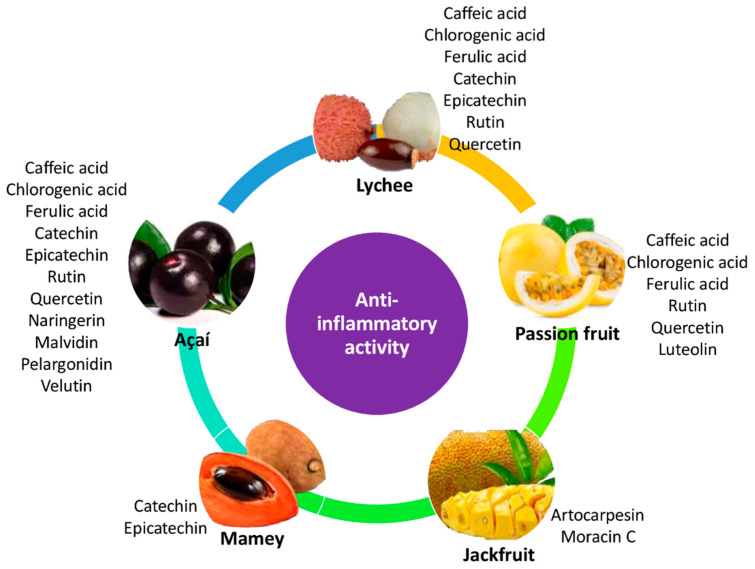
Main bioactive compounds present in lesser-consumed tropical fruits (lychee, mamey, passion fruit, açaí and jackfruit) known to exert significant anti-inflammatory effects.

Different mechanisms of action might be related to the anti-inflammatory effects of phenolic compounds. Among them, up/downregulation of transcription factors (e.g., NF-κB), inhibition of pro-inflammatory mediators (e.g., interleukin 6, IL-6), of activated immune cells (e.g., macrophages) and inducible nitric oxide synthase (iNOS) and cyclooxygenase-2 (COX-2), are considered in the present work and can be seen in Table 4 [80,81]. Figure 2 shows how several antioxidants, all found in lesser-consumed tropical fruits, can mitigate or even stop several pathways that lead to inflammation. The possible mechanisms of this action are not yet clear; however, they can inhibit the cascade of pro-inflammatory signals, such as caspases, COX-2, iNOS, IL-6, TNF-α, among others [86,87,88]. Figure 3 summarizes the main bioactive compounds in lesser-consumed tropical fruits, which are known to have significant anti-inflammatory activity.

#### 5.2.1. Changes Exerted by Altering Gene Expression

Moracin C is a phenolic compound found in jackfruit, which can significantly inhibit the release of ROS and lipopolysaccharide (LPS)-induced nitric oxide in RAW 264.7 cells, at doses of 25 and 50 μM without apparent cytotoxicity after treatment for 48 or 72 h. Regulation of the expression of iNOS, COX-2 and pro-inflammatory cytokines (IL-1β, IL-6 and TNF-α) was observed in response to these treatments. The anti-inflammatory action of moracin C was associated with the activation of some MAPK, including p38, ERK, and JNK, and NF-κB pathways [87]. Anti-inflammatory activity of phenolic compounds artocarpesin, norartocarpetine and oxyresveratrol isolated from jackfruit was reported. Artocarpesin suppressed LPS-induced nitric oxide and prostaglandin E2 (PGE 2) by downregulating the expression of iNOS and COX-2 [86].

Açaí pulp extract has anti-inflammatory properties that strongly inhibit COX-1 and COX-2 in vitro. It has also been shown to protect umbilical vein endothelial cells (HUVEC) against glucose-mediated inflammation by reducing the expression of IL-6 and IL-8 [89,90]. Velutin is a flavone in açaí pulp that inhibits NF-κB activation induced by oxidized LDL (oxLDL) in RAW-Blue cells. Its inhibitory effect was better than luteolin, another flavone with high anti-inflammatory activity [91]. The chemical structures of both compounds are similar, differing only in two substituents, where velutin bears two methoxyl groups at 7- and 3′-positions, whereas luteolin has two hydroxyl groups. Substitution of methoxyl groups appears to be a significant factor that regulates some bioactivities of the molecules, as determined by the evidence described. Velutin has also been shown to reduce the production of TNF-α and IL-6 in peripheral murine macrophages andactivate NF-kB [89,90].

A flavanol-rich lychee fruit extract (FRLFE) has been reported to contain a mixture of oligomerized phenolic compounds rich in monomers, dimers, and trimers of flavanol. Supplementation with FRLFE has been shown to suppress inflammation and tissue damage, both caused by high-intensity physical training of young long-distance runners for two months. Treatment with FRLFE reduced the serum concentration of IL-6 and significantly increased the transforming growth factor-β level between pre- and post-training [92]. Additionally, its effects on the expression of inflammatory genes were observed in rat hepatocytes treated with IL-1b and decreased mRNA and protein expression of iNOS, leading to an inhibition of nitric oxide and IL-1β. FRLFE also inhibited phosphorylation of the NF-κB inhibitor (IκB-a) and reduced the mRNA expression of NF-κB and TNF-α [93].

A lychee seed extract (LSE) rich in rutin, scopoletin, cianidanol, procyanidin D, phlorizin, 3,5-Dihydroxy-benzoic acid and 3,4-Dihydroxy-benzaldehyde demonstrated to decrease mRNA expression of NF-κB and apoptosis regulator Bax in eighteen diabetic male Sprague Dawley rats with induced hepatic injury indicating that the daily administration of 30 mg/kg of LSE for 6 weeks could avoid hepatic damage and diminish inflammation through these pathways [94].

Flavonoids and ferulic acid found in passion fruit flour have been shown to improve obesity-related inflammation, according to a decrease of TNF-α and IL-1β and inactivation of JNK. Results were found in male Sprague Dawley rats fed with a high-fat diet, with 50% of the cellulose replaced by *Passiflora edulis* peel flour. This suggests that these compounds protect from obesity-related inflammation; thus, passion fruit flour containing 60.9% of total fiber (19.94 soluble fiber and 40.15% insoluble fiber), 137 mg/100 g dw of total carotenoids, 116 mg catechin equivalents (CE)/100 g dw of total flavonoids and 14 mg/100 g dw of ferulic acid could mitigate this condition in obese subjects [88].

#### 5.2.2. Changes Exerted by Targeting Different Metabolites

Other compounds from jackfruit significantly inhibited the release of β-glucuronidase and histamine from P-methoxy-N-methylphenylethylamine (dihydroisocycloartomunin), inhibited lysozyme release (artocarpanone), and the formation of superoxide anion (cycloheterohyll, artonins B and artocarpanone) in rat neutrophils stimulated with formyl-Met-Leu-Phe (fMLP), as well as the inhibition of nitric oxide production and iNOS expression in RAW 264.7 cells (artocarpanone) [95].

**Table 4:** Bioactive compounds from mamey, açaí, passion fruit, lychee, jackfruit, and their anti-inflammatory activity.

Source	Compound	Classification	Activity	Reference
Açaí	Velutin	Flavone	Inhibit SEAP secretion Inhibited the expression of TNF-α and IL-6	[91,96]
Açaí pulp	Anthocyanins cyanidin-3-rutinoside and cyanidin-3-glucoside	Anthocyanins	↓ IL-6 and IFN-γ	[47]
Açaí seed extract	Catechin, epicatechin, and polymeric procyanidins	Polyphenols	↓ NF-κB and IL-6	[25]
Lychee	Catechin-type monomers and oligomers of proanthocyanidins	Flavanols and proanthocyanidins	Suppression of NF-κB activation and ↓ IL-6 and TNF-α	[97]
Lychee seed extract	21 compounds, including 3,5-dihydroxybenzoic acid, 3,4-dihydroxybenzaldehyde, procyanidin D, cianidanol, cinnamtannin B1, procyanidin A1, scopoletin, rutin, phlorizin and epicatechin–epicatechin– catechin	Polyphenols	↓ mRNA levels of NF-κB	[94]
Passion fruit peel flour	Vicenin, isoorientin, orientin, vitexin and isovitexin	C-glycosyl flavonoids	↓ IL-1β, Il-6 and IL-17	[20]
Purple passion fruit peel	Quercetin, luteolin, cyanidin 3-*O*-glucoside	Flavonoid	↓ NO levels	[98]
Yellow passion fruit peel flour	Ferulic acid	Hydroxycinnamic acid	↓ Lipid peroxidation ↑ GPx and GR in liver ↓ TNF-α and IL-1β Inactivation of JNK	[88]
Jackfruit	Artocarpesin	Flavone	Suppressed LPS-induced production of NO and PGE2, by downregulating inducible iNOS and COX-2 protein expressions	[86]
Jackfruit	Moracin C	Arylbenzofurane	Inhibited LPS-activated ROS and NO release, ↓ mRNA and protein expression of iNOS, COX-2, IL-1β, IL-6 and TNF-α	[87]

SEAP: Secreted embryonic alkaline phosphatase; TNF-α: tumor necrosis factor-α; IL-1β: interleukin-1β; IL-6: interleukin-6; IFN-γ: interferon-gamma; PGE_2:_ prostaglandin E_2._ GPx: glutathione peroxidase activity; GR: glutathione reductase; iNOS: inducible NO synthase; 12-HHT: 12(S)-hydroxy(5Z,8E,10E)-heptadecatrienoic acid; TXB2: thromboxane B2; 8- iso-PGF2α: 8-iso-prostaglandin F2α; 8-OHdG: 8-hydroxy-2′deoxyguanosine; MDA: malonaldehyde; JNK^MAPK^: c-Jun N-terminal kinase; COX-2: cyclooxygenase-2; IP-10: interferon gamma-induced protein-10; MCP-1: monocyte chemotactic protein-1; ICAM-1: intercellular adhesion molecule-1; VCAM-1: vascular cell adhesion molecule-1; MRP1: multidrug resistance protein 1.

Passion fruit peel flour treatment (8 mg/mL in the drinking water) has shown an anti-inflammatory effect on the intestine of female C57BL/6J mice by attenuating colitis-induced damage. Biochemical and molecular analyses revealed the inhibition of the expression of pro-inflammatory cytokines and an improved intestinal protective barrier. In addition to these effects, increases in the formation of short-chain fatty acids were observed, which are known to play an important role in the maintenance of colonic homeostasis; any imbalance in the microbiota homeostasis can up-regulate the immune response leading to mucosal damage and intestinal inflammation, thus supporting a prebiotic effect of passion fruit peel flour [20].

Different treatments can reduce or inhibit several inflammation cascades based on the bioactive compounds found in the lesser-consumed tropical fruits and their by-products. Nevertheless, further research is still needed to identify, isolate, and quantify their phytochemical content and determine their efficacy on human health.

## 6. Conclusions

The present review brings attention to the phytochemical content of lesser-consumed tropical fruits such as passion fruit, lychee, mamey, açaí and jackfruit, as well as their by-products. Their phytochemical composition is associated with some biological activities, such as antioxidant and anti-inflammatory activities. Particular compounds such as velutin, moracin, malvidin, pelargonidin, naringerin, ferulic acid, chlorogenic acid, caffeic acid, catechin, epicatechin, quercetin and rutin found in these fruits are promising candidates for developing novel functional foods and/or nutraceuticals. However, their safety must be validated, while additional in vivo trials are required as a precedent to their pharmacological capitalization in contemporary medicine. Detailed studies on their pharmacokinetic behavior are also lacking, and these are required to associate their consumption with specific biological activities conclusively and indisputably be at the heart of upcoming research. Additional data generated on the lesser-consumed tropical fruits will serve to increase their production and consumption in diverse markets. At the same time, research into their by-products will allow their incorporation into the circular bioeconomy.

## Data Availability

Not applicable.
